# The University of Limerick Education and Research Network for General Practice (ULEARN-GP): practice characteristics and general practitioner perspectives

**DOI:** 10.1186/s12875-020-1100-y

**Published:** 2020-02-05

**Authors:** Andrew O’Regan, Peter Hayes, Ray O’Connor, Monica Casey, Pat O’Dwyer, Aidan Culhane, Patrick O’Donnell, Gary Stack, John Cuddihy, Billy O’Connell, Jerry O’Flynn, Walter Cullen, Jane O’Doherty, Maurice O’Connell, Liam Glynn

**Affiliations:** 1 Graduate Entry Medical School, Faculty of Education and Health Sciences, University of Limerick, Limerick, Ireland; 2 Health Research Institute, University of Limerick, Limerick, Ireland; 3 School of Medicine, Health Sciences Centre, University College Dublin, Belfield, Dublin, Ireland

**Keywords:** General practice, General practice research, Practice-based research networks, General practitioners, Primary healthcare, Health systems research

## Abstract

**Background:**

A well-functioning general practice sector that has a strong research component is recognised as a key foundation of any modern health system. General practitioners (GPs) are more likely to collaborate in research if they are part of an established research network. The primary aims of this study are to describe Ireland’s newest general practice-based research network and to analyse the perspectives of the network’s members on research engagement.

**Method:**

A survey was sent to all GPs participating in the network in order to document practice characteristics so that this research network’s profile could be compared to other national profiles of Irish general practice. In depth interviews were then conducted and analysed thematically to explore the experiences and views of a selection of these GPs on research engagement.

**Results:**

All 134 GPs responded to the survey. Practices have similar characteristics to the national profile in terms of location, size, computerisation, type of premises and out of hours arrangements. Twenty-two GPs were interviewed and the resulting data was categorised into subthemes and four related overarching themes: GPs described catalysts for research in their practices, the need for coherence in how research is understood in this context, systems failures, whereby the current health system design is prohibitive of GP participation and aspirations for a better future.

**Conclusion:**

This study has demonstrated that the research network under examination is representative of current trends in Irish general practice. It has elucidated a better understanding of factors that need to be addressed in order to encourage more GPs to engage in the research process.

## Introduction

General practitioners (GPs) are situated at the front line of health care, where over 80% of all healthcare consultations take place [1], but practice involvement in research remains piecemeal [2]. In most countries, general practice has not engaged in research as much as other healthcare disciplines [3]. The Republic of Ireland, where this study took place, lags behind the UK in terms of investment in, and outputs from, General Practice research [4]. The reasons for this are unclear, but attempts to promote a culture of research continues to be hindered by the prevailing perception of research as a remote science and the absence of a supporting infrastructure [5–7]. Historically, general practice research has been under-resourced and marginalized [4, 8], but experts have agreed that the profession must progress as an academic discipline [9] in order to have evidence to inform decision making on issues that concern general practice [10]. Research that informs day-to-day practice [11, 12] and that benefits patients [13], attracts GPs who, in turn, get involved in studies that aim to improve population health [13, 14] and clinical management of patients [15]. Moreover, GPs are more likely to undertake research if they have been involved in research in the past [16], or if there is collaboration and support provided by a research network [17].

Practice-based research networks (PBRNs) have been defined as “collaborations between clinical practitioners and academics … to foster research in general practice through opportunities to learn more about how to undertake and participate in research, and assist in translating new knowledge into practice” [18]. They have been described as “a basic laboratory for primary care research and dissemination” [19]. PBRNs, through patient consent, can provide access to data on large numbers of patients [20], and can generate the capacity to investigate questions of importance to clinical practice, disseminate results, and implement evidence-based strategies [19]. Furthermore, they can act as a lever in improving primary care quality and outcomes as well as a driver for increased co-operation and collegiality among GPs [21]. By facilitating data pooling and the creation of large clinical databases [22], they can provide important clinical data for research questions relevant to day to day general practice [23], and have been successful in producing research outputs [24]. In Scotland, where there is a national PBRN, two thirds of practices are research-active and are highly productive [25].

Policy changes in medical education in Britain and Ireland have increased the primary care orientation of recent medical degree programmes [26, 27]. Furthermore, the concept of a longitudinal integrated clerkship (LIC) or extended placements in general practice is being adopted more widely [28]. Ireland’s newest medical school, the Graduate Entry Medical School at the University of Limerick was established in 2007. As part of their clinical training all medical students on the programme undertake an 18-week LIC in general practice which is unique on the island of Ireland. This presents an unprecedented opportunity for the teaching practices involved to work closely with a medical school over a continuous period in time. The educational benefits have been described [29, 30], but the research potential of such a relationship has not been investigated.

The primary aim of this study is to describe Ireland’s newest general practice-based research network and to examine the research priorities of the GPs within the network and the barriers and levers to engagement with a research agenda in everyday practice.

## Method

### Setting

**Fig. 1:**
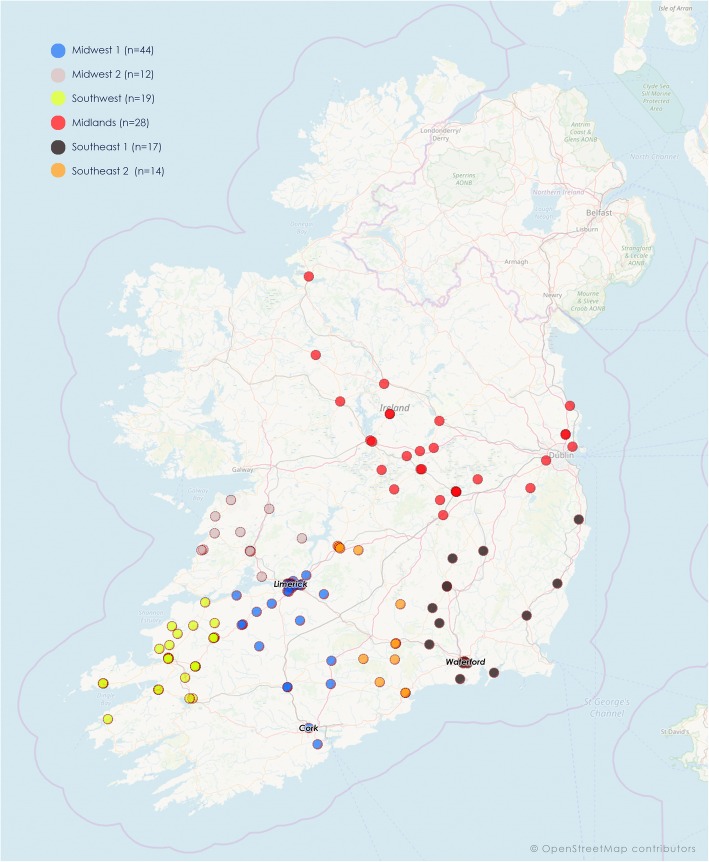
Location and distribution of ULEARN-GP affiliated practices. Each pin represents a single practice. They are colour-coded by regional hub

The University of Limerick General Practice Education and Research Network for General Practice (ULEARN-GP) currently consists of 134 general practices distributed across the Republic of Ireland. Figure 1 outlines the geographic distribution of the practices in the ULEARN-GP network which is now a national network.

The ULEARN-GP practices are located in all four of Ireland’s Health Service regions. The network is organised into six regional ‘hubs’. These are: South West, Clare, Limerick, South-East-1, South-East-2 and Midlands. Each hub is led by a GP co-ordinator, with students attending a local teaching ‘hub’ for 1 day of formal teaching for each week of their 18-week LIC placement in general practice. The core teaching activity of this placement is ‘parallel consulting’, whereby students see patients initially on their own followed by the opportunity to present, examine, diagnose and treat under the supervision of their GP tutor. Students are also encouraged to engage in all the routine clinical activity of the practice as well as research and audit. The school had its first intake of medical students in 2007 and this class graduated in 2011.

Primary care in Ireland comprises of a mixture of public and private healthcare models, whereby approximately 40% of the population (people with certain chronic illnesses and all those below certain income thresholds) have their medical care paid for by the General Medical Services (GMS) scheme and do not pay for their GP visit or prescribed medications [31]. A second cohort receive a doctor visit card and do not pay for GP visits but must pay for medications. GPs receive an annual payment for taking care of patients under the GMS scheme and with doctor visit cards [32]. Private patients pay a fee of approximately €50/60 euro per visit.

### Quantitative data collection

A profiling survey questionnaire was posted and e-mailed to all practices affiliated with the University of Limerick Graduate Entry Medical School. The questionnaire was a modified version of that used in a similar study [33] – see Additional file 1. The profiling questionnaire used in the study gathered demographic details on practices, including details on practice staff, organisation, size and academic activity. Descriptive statistics on practice descriptors were analysed using SPSS version 24. These figures were then compared with other published profiles of Irish general practice [34].

### Qualitative data collection

GPs affiliated with the University of Limerick-Graduate Entry Medical School were invited by email to participate in an interview by email in December 2016. A purposive sampling methodology was chosen in order to involve participants that were known to be key rich informants [16]. This method also facilitated the recruitment of a sample of GPs with varying personal and practice characteristics, including years of experience, involvement in postgraduate education, practice size and location. Table 1 outlines the characteristics of the GPs interviewed. The interview guide (Additional file 2) was developed by a number of the authors with experience in qualitative methods and with research activity in general practice, and was piloted and subsequently adapted before the study began. Interviews (duration from 20 to 68 min) were conducted by the first author (AOR), an experienced qualitative researcher and GP, who had no relationship with the participants at the time. Each interview was digitally recorded, either at the participants’ clinics or in the university, and field notes were taken. All recorded interviews were transcribed verbatim (denaturalized transcription), and anonymised through the removal of all identifying information. In line with the simultaneous analysis and collection of data that is an integral part of qualitative analysis, the interview guide was modified as the transcripts were reviewed to reflect the emergence of new themes and nuances highlighted in previous interviews. Sampling continued until data saturation had been reached to the extent that the data that had been collected and analysed were sufficient to address the research question and provide a variation of experiences.

**Table 1:** GP participant demographic details

Years of general practice experience	Practice type	Practice location	Involved post graduate GP training
< 10 years (*n* = 1)	Single handed (*n* = 5)	Urban (*n* = 10)	Yes (*n* = 11)
10–20 years (*n* = 3)	2–3 GPs (*n* = 8)	Rural (*n* = 6)	No (*n* = 11)
> 20 years (*n* = 18)	> 3 GPs (*n* = 9)	Mixed (*n* = 6)	

Thematic analysis was carried out using NVivo software (version 11) following a thematic process outlined by Braun and Clark [35]. As such, the “keyness” of a theme was not necessarily dependent on quantifiable measures, but in terms of whether it captured something important in relation to the overall research question. Similar themes from each transcript were identified and grouped and then overarching categories were identified through examining the relationship between the themes. Final themes were agreed between four authors (AOR, MC, JOD, ROC) and the senior author audited the final analysis.

Ethical approval was granted by the Education and Health Sciences Research Ethics Committee, University of Limerick. GPs were not offered any incentives to participate.

## Results

### The ULEARN-GP network profile

Table 2 describes ULEARN-GP member practices and compares them to the national profile as described by data from other published research networks. Thirty-five per cent were rural, 18% were urban and just under half the practices were mixed. All of the practices were computerised, with 89% using Socrates or Health- One. The median number of GPs working in each practice was two and 15% were single-handed. Over half of the practices had three or more GPs, while 32% had three or more full time equivalent GPs. One practice did not have a practice nurse and nearly half the practices had more than one nurse. One practice did not have administrative staff with 80% having more than one administrator and 16% employing a practice manager. All of the practices operated a form of shared out of hours system, with 94% of practices involved in an out of hours co-operative and 6 % opting for a privately funded locum system. Three quarters of GPs were coding chronic disease, with 17% coding individual consultations. International Classification of Primary Care (ICPC) was used slightly more than International Statistical Classification of Diseases and Related Health Problems (ICD-10). Sixty-two per cent were involved in research in the preceding three years.

**Table 2:** Comparison of National, West of Ireland Research and Education Network (WestREN) and ULEARN-GP profiles

	National 1992 [34]	National 2005 [34]	WestREN 2009 [33]	National 2015 [34]	ULEARN-GP 2018
No. of practices	428	545	71	462	134
Response rate	68%	87%	73%	72%	100%
Practice Type					
GMS() + Private	91%	96%	100%	89%	100%
Private practice only	9%	4%	0%	11%	0%
GMS List Size					
< 500	25%	27%	4%	18%	10%
500–1900	72%	71%	82%	75%	58%
> 2000	3%	2%	14%	7%	32%
Practice Location					
Rural location	33%	21%	50%	21%	34%
Urban location	47%	43%	25%	42%	18%
Mixed location	20%	36%	25%	37%	48%
Premises					
Purpose built premises	27%	43%	54%	54%	37%
Adapted premises	46%	46%	40%	43%	53%
Attached to residence	27%	11%	5.7%	3%	1%
Practice Organisation					
Computerisation	27%	89%	100%	94%	100%
Out of Hours					
Internal rota	25%	5%	4%	1%	0%
External rota	60%	15%	19%	6%	6%
Co-operative	0%	42%	61%	93%	94%
Practice staff					
Single handed GP	57%	35%	25%	18%	15%
Practice nurse	17%	75%	92%	82%	99%
Education					
Involved in postgraduate training	8%	18%	42%	22%	63%

### Qualitative evaluation of research priorities, barriers and levers

Twenty-two GP participants were invited to participate and all agreed to be interviewed.

Four major themes summarised below in Table 3 emerged from the qualitative data and these were: catalysts; coherence; systems failure; and aspirations for a better future. Major themes were divided into a series of subthemes described below which were often inter-related.

**Table 3:** Summary of qualitative data in major themes and subthemes

Major theme	Subtheme
Catalysts	Evidence-based practice and practice-based evidence
	Organisational catalysts
	Students as catalysts
Coherence	Coherence in policy and academia
	Novice mentality
	Normalisation
Systems failure	Boundaries
	Research culture
	Scaffolding and support
Aspirations for a better future	Partnership and equity
	GPs fulfilling potential
	Foundations for a research network

### Catalysts

‘Catalysts’ describes an overarching theme of concepts and factors that GPs perceived to be important to promote participation in research. It contains the following subthemes: evidence-based practice; practice-based evidence; organisational catalysts; and student interaction. These subthemes describe the key motivational context and interactions within the working environment that have the potential to stimulate research activity.

### Evidence-based practice and practice-based evidence

GPs universally appreciated the importance of basing their clinical practice on sound principles grounded in evidence. When GPs recognised the relevance of research to how they practise medicine and to their patients, they see a value in it. A very strong patient-centred theme was present throughout the interviews. The role of general practice and the research conducted within it should be to produce better health services and outcomes for patients.

*“I believe research in general practice should be very much focused on the pragmatic stuff, on the delivery of care, on how we can best deliver care to our patients. And where there is an evidence base as well … It is about improving care for the patients. That is what it is about.”* GP 6

The data clearly described the evolution of the clinical role of the GP from treating acute and often minor illnesses to managing chronic illnesses, preventative medicine and an overall increase in complexity. This development was viewed as an impetus for research in general practice so that the management of patients with these conditions would be informed by up-to-date evidence which would emerge from primary care settings. There was interest among GPs in directing research that would answer clinical questions to inform guidelines and provide an evidence-base from-community-based research. In addition, many participants articulated the need for GPs to contribute to formulating research agendas and setting research questions.

### Organisational catalysts

Health services research, specifically examining the processes of care and how health care is delivered, emerged as a strong area of interest from the data. GPs were aware of the impact of their work on the patients and community they serve and, specifically, how their work fits in the context of the wider health service. They recognised that the computerised data collected through their daily clinical practice has the potential to contribute important data to research of health care delivery. The wider political context of health service restructuring, ongoing GP industrial relations issues and the decline of rural general practice emerged as significant sources of concern for GPs. Participants voiced a degree of curiosity about their own performance and the hidden value of the work that they do.

*“the patchwork quilt type of quality of what happens between doctor and patient … I would love to see more of the technicolour aspect of what actually goes on in the consultation.”* GP 19

### Students as catalysts for research

For many GPs, interaction with medical students on placement, who had research and audit requirements of their own, was an important introduction to having research conducted in their practices. Perceived benefits of a student in the practice included stimulating reflection on their practice, the role of the student in collecting data and students presenting findings back to the GPs and staff in the practice. In this context, the student was perceived as a bridge between practices and the medical school and, in some instances, were seen as an educational source for GPs on research methods. Some participants discussed examples of projects, designed by faculty, whereby students could opt in and receive training on data extraction using practice software to answer a specific research question. In such instances, the stages of getting ethical approval, developing a coding sheet and piloting of the study were carried out by faculty. The GP tutor would then collaborate with the student on placement and many found it useful as a way of understanding the research process and learning how to use the practice software for data collection purposes.

*“Certainly, student projects have helped me re-examine my own practice. I can look at it and say ‘why do I do it that way’ or ‘I didn’t realise that was going on”* GP 3

### Coherence

Coherence refers to the multi-faceted journey for GPs in making sense of the relevance of research to them. Coherence in this context includes the following subthemes: coherence in policy and academia, novice mentality relating to research among GPs and normalisation of research as part of general practice.

### Coherence in policy and academia

GPs perceived a lack of consistency of priorities among the principle health service bodies, as well as a perceived disconnect between medicine and research in the hospital and medicine and research in the community. A clear perception emerged from the data that academic bodies have not respected them, have not valued their input into research and that their data is used but they are not approached for intellectual input, they are not acknowledged or rewarded for their contribution.

*“It is again just about the whole remove of academia, and the idea that they talk about research in general practice. You know, it is like apples and oranges. They think in a different reality*.” GP 12

### Novice mentality

GPs perceived that they do not have the expertise or experience to formulate research questions or to get started on a research study. Many were working in isolation and lack the encouragement and support that is needed to undertake a research study. There was awareness of the steps involved in conducting studies. However, lack of confidence in their ability as researchers was a recurring theme in the data. It seemed that GPs viewed that research as an activity for other professionals.

*“people think research is [what] people with white coats do a lot and it’s very remote and irrelevant and a lot of the stuff that you actually see in the so called high powered peer reviewed journals, a lot of it is of little relevance to guys … in the trenches.”* GP 19

### Normalisation – research as part of the GP role

This theme refers to research becoming a normal part of practice. Notwithstanding the lack of consistency in policy around research and the poor self-image of GPs as researchers, most participants expressed a vision of how research could become embedded in practice. GPs realised the importance of the data that they had collected in practice computers and were aware of the need for other research team members such as statisticians and qualitative experts. In several interviews, although the participants initially spoke about their lack of time and interest in research, later they would express a vision of how research could happen in general practice, the support that would be needed and how they themselves might contribute. They saw their role not as static but having the potential to change.

*“It is not everybody’s cup of tea and you can’t expect it to be...and you will have people who will be very happy to be data collectors but who don’t want to be involved in the nitty gritty writing up of the (project).”* GP 14

### Systems failure

The third overarching theme described how participants felt that the current health system is destined to fail with regard to the production of general practice-based research. Subthemes described the problems with the system that prevent GP engagement such as a lack of boundaries around the role of the GP; lack of research culture and lack of scaffolding and supports.

### Boundaries

The ever-increasing workload for GPs, coupled with lack of time for pursuing activities other than service provision were identified at an early stage in the data analysis as major and recurring barriers to research activity. On further analysis, lack of clear boundaries around the role of the GP emerged as the most important contributing factor. The way the system is designed currently means that GPs are being tasked with increasing responsibilities and associated administration and are not in a position to refuse any of the demands being placed on them. Poor health service planning, with no clear vision of the potential of general practice and how it can be supported is a significant part of the problem.

*“Whenever there is some hassle out there in the health services, someone up in the offices says “sure look, the GPs will take care of that, sure they always roll up their sleeves and get stuck into that”. That is kind of a general thing out there “your doctor will always take care of it”. But, you see, we don’t have protected time to do anything else other than keep our head down to the grindstone practising medicine.”* GP 9

### Research culture

Relating to the lack of boundaries in the system, a culture appears to have developed whereby GPs, their peers and the wider health system do not value the potential contribution of GPs to research. When research is not prioritised and an attitude of passivity or disinterest develops, research is seen as a burden rather than a natural component of the role that could enhance the working life of a GP. There was a sense of despair with the current working environment coupled with a suspicion of research proposals.

*“I think people are frightened and I think that general practice sees it as a bit of a burden. Like don’t ask us to do anything else. You know, it is their only way of showing that they are suffering ‘don’t ask me to do anything more. I am fed up and I am tired and I don’t want to know about it’. They see it as the business for men in suits”* GP 14

### Scaffolding and supports

Participants discussed the lack of infrastructure or readily available expertise from their own peers and from academic bodies with which they could discuss and develop ideas. Similarly, the paucity of readily available frameworks and training in research mean that research is unlikely to grow in general practice in the current climate.

*“You could spend hours yourself trying to look at ways of doing it but, if they could send someone that could take a couple of minutes to look at it and answer the questions for you, that would be very helpful.”* GP 21

### Aspirations for a better future

The first three themes all contribute to a system that seems to be poorly set up and under resourced. Despite these negative factors, many participants had very clear ideas on how things could improve. This fourth and final major theme describes the GPs aspirations for a better future in terms of research engagement, and that they saw a place for the network in improving research engagement and ultimately the care of patients. The subthemes here, included partnership and equity; fulfilling potential; and foundations for a research network.

### Partnership and equity

The medical school must work to develop relationships with GPs, to build trust and mutual respect and take measures to include GPs in the academic environment. Some of those interviews described previous negative experiences when they had collaborated by academic bodies by facilitating data collection from their software and were never properly acknowledged for their input.

*“If GPs felt more included and associated more with the medical school’s events, they might feel more of an urge to meet other GPs and collaborate on various research projects.”* GP 12

### GPs fulfilling potential

The health services and other bodies in the health system can support increased GP involvement by organising and financing protected time for the GPs, and by providing resources such as computers and trained staff such as research assistants. Furthermore, for GPs with a serious academic interest, there could be the possibility of resourcing them to take time out and go into the universities for training and meetings.

*“Facilitate his involvement by buying the time out of his practice that would cost money and provide research assistants that would add to the practice for access to his data. By giving something back to the practice, by giving him suggestions on care for patients with certain conditions and suggestions for improving care and if he does that then his name goes on papers and being part of a research group.”* GP 8

### Foundations for a research network

From the data a new spectrum of possible relationships between academia and GPs can be seen. This ranges from one-to-one to an active forum of peers; to establishing a full research network of GPs. This is the new infrastructure that could be built on the principles outlined. In order establish and sustain this, leadership is required.

*“I have this concept of the unseen university … it is almost like a hedge school, you don’t have to have a big building to have a university. It can be all scattered throughout the country. And this is a kind of a network of practices... It is like what in Natural History we used to call “field research”. Instead of having all the animals inside in a lab running around you actually go out there with your Land Rover and camera and see what they are actually doing.”* GP 4

## Discussion

### Summary of main findings

This study has described the demographics of the ULEARN-GP network, Ireland’s newest practice-based research network, and it has shown that it is representative of trends in Irish general practice nationally. Comparison has been made to four previous surveys of Irish general practice, with some interesting patterns emerging. Notably, national shifts towards larger practices, more part-time work and a decline in both single-handed and rural general practice are reflected in the profile of ULEARN-GP network. In addition, this study highlights the barriers that exist and potential solutions to fostering a culture of research in practice-based research networks.

### Comparison to existing literature

Rural and remote GPs in Canada have described capacity as well as attitudinal barriers to research engagement [36]. The shift towards larger practices is reflective of international trends towards fewer but larger practices that may amalgamate to form networks [37]. Smaller practices have unique needs and priorities which inhibit research activity [38]. The discrepancy between practices with three or more GPs (one half of all practices) and three or more full time equivalent GPs (one third of all practices) reflects the shift towards more part-time work, which may negatively affect research potential. It is encouraging that almost two-thirds of practices had participated in research in the past 3 years, most likely through students carrying out research projects under the joint supervision of faculty and the GP tutor. An urban-rural mix and computerised practices are factors that promote research in PBRN [39] which bodes well for the network under study.

The GPs in this network appeared to recognise the importance of, and have an interest in, research in general practice. Increased opportunities for research involvement may, therefore, influence research activity and capacity but such opportunities remain dependent on factors such as training, protected time and funding. The participants valued the impact of relevant research, evidence-based practice and practice-based evidence, in keeping with previous studies [12, 40]. Study participants expressed the need to systematically document the work GPs do in order to influence policy; a point also made in a report on general practice in the UK [41].

Practice characteristics are changing and this is seen in the increasing proportion of practices of over 2000 GMS patients. While the percentage of practices with very large GMS sizes is higher in the ULEARN network compared to the most recent national figure, the overall trend is towards larger sizes. Premises that were attached to private residences accounted for one quarter of practices in 1992 and the proportion has steadily declined to 1%. The trend towards increased computerisation and appointment systems has reached 100%. Co-operatives have replaced rota systems with 94% of practices opting for that route. In 1992, over half of practices were singlehanded and this has declined steadily to 15%. Meanwhile, the proportion of practices involved in postgraduate training nationally is growing but the proportion involved in this activity among the WestREN and ULEARN-GP networks is much higher.

Individual barriers to research activity described in this study have been previously reported, such as lack of time [13, 42, 43], clinical workload [44] and inadequate training in research methodology for GPs [43, 45, 46]. It became clear from the analysis of the data that these individual factors were common to all and are, in reality, part of a systems malfunction in the health service. This is a multifaceted problem and involves: lack of boundaries regarding the role and extent of general practice; lack of understanding of the potential of general practice to contribute to research; lack of coherence between the stakeholder bodies and disconnect between academia and practice and hospital research and its implementation in the community.

Strategies identified by GPs in this study to enable future research involvement included: collaboration with other health organisations; involvement of professional researchers at all stages, particularly by developing relationships between GPs and the university through ULEARN-GP. Similarly, GPs in the UK [47] and nurses in Northern Ireland [48] prioritised organisational infrastructure and an appropriate working environment in developing research capacity as opposed to personal skills and attributes which were ranked relatively lower in importance. The Mant report [4] confirmed the under-performance in terms of output in Irish primary care compared to the UK despite the fact that a similar proportion of primary healthcare professionals appear to be involved in research in both settings. Such a discrepancy suggests that organisational and cultural factors must be addressed. Some research experts have called for caution in the drive for research productivity, pointing to a paucity of high-quality studies generated by PBRNs over three decades in the USA [49]. On the other hand, its grounding in day-to-day practice [50] makes general practice a source of very rich data.

Support from local academic departments is important factor in developing networks of teaching and research practices, in order to provide infrastructure for academic activity [40]. An example of the support may be the provision of a suitable research methodology course by universities, an initiative shown to improve research activity among GPs [51]. Training and close collaboration with academic faculty can enable GPs to become more involved in research projects [17], as well as creating academic positions for GPs and the appointment of research fellows [39]. The importance of partnerships between professionals and professional bodies to enable future activity has been reported [52]. Government investment in supporting academics and supporting establishing PBRNs are necessary in order to build research capacity in primary care [53]. Levers for overcoming the barriers of time and skills capacity, include buying GPs out of clinical time to engage in research and to partner with academics for research training [54].

This study shows how misperceptions about research and the disconnect between clinicians and academia were part of a negative cultural trend that kept general practice and research apart. This is not unique to the Irish context, with GPs in France perceiving medical research as the scope of a “laboratory worker” [50] and another study citing negative experiences with research creating distrust within the profession [45]. The term ‘organisational coherence’ has been previously used in this context to describe how PBRNs can develop synergy with other local services and generate “an environment for cross pollination of ideas and sharing of organisational structures” [55]. Thomas described how PBRNs should operate as “university-linked localities”, becoming a community of practice whereby clinical and academic expertise can improve health services locally [56]. PBRNs can bridge the gap between science and implementation, research and practice [57]. Furthermore, research has reported how organisational collaboration for research through PBRNs can lead to clinical primary care networks [21]. Ultimately, the aim is to conduct research that is relevant in collaboration with others that will improve health care [58].

### Strengths and limitations

The strengths include that this paper presents the results of a survey of a large practice-based network with a high response rate, which indicates enthusiasm among stakeholders. In addition, the qualitative data sample was large (*n* = 22) and achieved data saturation. The research team comprised of clinical and scientific researchers which enriched the interpretation of data. The interviews were conducted by a GP which enabled clarification of clinical language and encouraged participants to speak about their personal experiences. Limitations are that the sample was only representative of GPs from Ireland and only GPs affiliated with the medical school were recruited. While practices will have accurate numbers of public patients, it is difficult to establish accurate numbers of private patients as they do not have to register with a single GP and may attend for once off visit and be counted among the practice population- this may over or under estimate practices sizes.

### Implications for future research and practice

Developing ULEARN-GP into an effective vehicle for community-based research and dissemination is a priority. The experiences of stakeholders, as well as processes and outcomes will be investigated over time. The practice characteristics described in this study are representative of national trends in terms of size, location, organisation and infrastructure. This study has deepened the understanding of factors that need to be addressed in order to encourage a cultural shift in thinking about primary care research for GPs, specifically, clarifying and protecting the role of the GP, as well as closer collaboration with academic institutions. Despite the challenges, GPs, given the appropriate environment, are willing and able to engage in research activity. However, it is clear from the data that in order for GPs to engage in a meaningful way with the research process, their input must be valued. This will require a shift in thinking about the academic potential of GPs by health service planners as well as funding bodies, whereby the contribution of GPs is recognised and understood and GPs are paid appropriately for their time. This could involve the health service paying GPs for academic sessions or creating more part-time academic posts for clinical academic GPs. Finally, this study also helps to identify a population of professionals for any future research strategy, thus increasing the likelihood of any such research strategy achieving enduring success.

## Conclusions

This study has compared the characteristics of the country’s newest General Practice research network and has demonstrated that it is representative of current trends in Irish general practice. It has elucidated a better understanding of factors that need to be addressed in order to encourage more GP’s to engage in the research process.

## Supplementary information

**Additional file 1:** Survey sent out to ULEARN-GP practices May 2018.

**Additional file 2:** Interview guide.

## Data Availability

Data is available by reasonable request to the corresponding author.

## Abbreviations

GP: General practitioner
PBRN: Practice-based research network
ULEARN-GP: The University of Limerick Education and Research Network for General Practice
