# Supplementary material for: The University of Limerick Education and Research Network for General Practice (ULEARN-GP): practice characteristics and general practitioner perspectives
Source: BMC Fam Pract. 2020 Feb 5;21:25. doi: 10.1186/s12875-020-1100-y (PMC7003418; doi:10.1186/s12875-020-1100-y)
Supplement: Supplementary file 1 — Additional file 1: Survey sent out to ULEARN-GP practices May 2018. [file 12875_2020_1100_MOESM1_ESM.docx]

**Survey sent out to ULEARN-GP practices May 2018**

Q1 Name of practice

Q2 Type of main practice premises (please tick all that apply): purpose-built; adapted; attached to home; co-located with HSE

Q3 Appointment systems (please only choose one); appointment only; walk-in only; mixed appointed and walk-in sessions

Q4 Are computers used in your practice? (Please tick only one): Yes; No

Q5 If so to what extent are they used? (Please tick as appropriate): full for all consultation notes, prescriptions and patient correspondence; partial

Q6 If partial (please tick all appropriate options): Repeat prescriptions, consultation notes, outgoing

referral letters/correspondence; scanning incoming correspondence, Healthlink, e-referral.

Q7 Practice software package (please tick one): Socrates; GP Dynamic; GP Mac; Helix Practice Manager; Health One; Other (please specify)

Q8 Do you code chronic diagnoses for each patient (e.g. diabetes/asthma)?: Yes; No

Q9 Do you code each consultation diagnoses for each patient (e.g. UTI)?: Yes; No

Q10 If so which coding system do you use? (please tick one or both): ICPC; ICD10

Q11 Number of individual GPs working within the practice?

Q12 How many FTE’s (full time equivalent GP’s. 1 FTE = 8+ clinical sessions per week)?

Q13 Does the practice have a nurse?: Yes; No

Q14 If yes how many full time equivalent practice nurses? (1 half day per week = 0.1 FTE).

Q15 Does the practice have administration staff?

Q16 Does the practice have a practice manager? : Yes; No

Q17 Total # GMS practice list

Q18 Total practice population

Q19 Weekly night duty (Mon-Fri inclusive): Average number of nights on call per 4 week period for the practice?

Q20 How many weekends per year is the practice on duty?

Q21 Is your practice attached to a GP training scheme? : Yes; No

Q22 In the past 3 years has this practice been a training practice for a GP training scheme? : Yes; No

Q23 In the past 3 years has the practice been involved in any general practice oriented research? : Yes; No

Q24 Are there any clinical dilemmas or research questions you personally would like to see the Network try to solve? If so please provide a brief outline below.

Q25 We would be delighted to hear any comments you have to make about being part of the U-LEARN GP (University of Limerick Education and Research Network)
