# Supplementary material for: The University of Limerick Education and Research Network for General Practice (ULEARN-GP): practice characteristics and general practitioner perspectives
Source: BMC Fam Pract. 2020 Feb 5;21:25. doi: 10.1186/s12875-020-1100-y (PMC7003418; doi:10.1186/s12875-020-1100-y)
Supplement: Supplementary file 2 — Additional file 2: Interview guide. [file 12875_2020_1100_MOESM2_ESM.docx]

**Interview guide**

1. Can you tell me about your experience with research as a general practitioner?

Prompts: have your participated in research in any way? Is research important for General Practice? Is it something you are interested in? Have you ever been asked to participate in research? Is research activity a priority for you as a GP?

1. What type of research do you think is relevant to General Practice?

Prompts: is some research more than others applicable to community settings? Are there research questions that arise during your day to day work? What is it about your practice that would be useful for answering research questions? What types of research questions and methodologies are you interested in?

1. What are the barriers to engaging more actively in research (for you and for GPs in general)?

Prompts: why are you not engaging in research daily? Have you ever had an opportunity to do research and been unbale? If there are questions that you and your colleagues would like to have researched what is stopping you researching them?

1. What do you see as principal strategies to engage GPs in research?

Prompts: locally is there anything that could be done to engage you and your colleagues? At a national planning level are there changes that could be made? Are there supports that if they were available would encourage you to become research active? What would have to change in General Practice infrastructure for you to become research active?

1. In an ideal world, how would you see the role of GPs in research evolving and being supported?

Prompt: ideally, what role would you like to play in research?

1. Is there anything further you would like to add?
